# Single-Dose Mucosal Immunization with a Candidate Universal Influenza Vaccine Provides Rapid Protection from Virulent H5N1, H3N2 and H1N1 Viruses

**DOI:** 10.1371/journal.pone.0013162

**Published:** 2010-10-04

**Authors:** Graeme E. Price, Mark R. Soboleski, Chia-Yun Lo, Julia A. Misplon, Mary R. Quirion, Katherine V. Houser, Melissa B. Pearce, Claudia Pappas, Terrence M. Tumpey, Suzanne L. Epstein

**Affiliations:** 1 Division of Cellular and Gene Therapies, Center for Biologics Evaluation and Research, Food and Drug Administration, Rockville, Maryland, United States of America; 2 National Center for Immunization and Respiratory Diseases, Centers for Disease Control and Prevention, Atlanta, Georgia, United States of America; IGMM CNRS 5535, France

## Abstract

**Background:**

The sudden emergence of novel influenza viruses is a global public health concern. Conventional influenza vaccines targeting the highly variable surface glycoproteins hemagglutinin and neuraminidase must antigenically match the emerging strain to be effective. In contrast, “universal” vaccines targeting conserved viral components could be used regardless of viral strain or subtype. Previous approaches to universal vaccination have required protracted multi-dose immunizations. Here we evaluate a single dose universal vaccine strategy using recombinant adenoviruses (rAd) expressing the conserved influenza virus antigens matrix 2 and nucleoprotein.

**Methodology/Principal Findings:**

In BALB/c mice, administration of rAd via the intranasal route was superior to intramuscular immunization for induction of mucosal responses and for protection against highly virulent H1N1, H3N2, or H5N1 influenza virus challenge. Mucosally vaccinated mice not only survived, but had little morbidity and reduced lung virus titers. Protection was observed as early as 2 weeks post-immunization, and lasted at least 10 months, as did antibodies and lung T cells with activated phenotypes. Virus-specific IgA correlated with but was not essential for protection, as demonstrated in studies with IgA-deficient animals.

**Conclusion/Significance:**

Mucosal administration of NP and M2-expressing rAd vectors provided rapid and lasting protection from influenza viruses in a subtype-independent manner. Such vaccines could be used in the interval between emergence of a new virus strain and availability of strain-matched vaccines against it. This strikingly effective single-dose vaccination thus represents a candidate off-the-shelf vaccine for emergency use during an influenza pandemic.

## Introduction

Pandemic influenza represents a major threat to global public health, with the potential for sudden emergence and explosive transmission of virus strains to which humans have little or no serologic immunity. Conventional influenza vaccines function by inducing antibodies against the highly variable surface glycoproteins hemagglutinin (HA) and neuraminidase (NA), and currently take at least 6 months to prepare and distribute once a potential pandemic strain has been identified [1], [2]. This was highlighted by the 2009 swine origin H1N1 pandemic virus: the newly emergent virus was identified in April but sufficient vaccine for mass immunization was not available until October. Meanwhile the virus was spreading in the community [3], [4].

So-called “universal” influenza vaccines providing cross-protective immunity in a strain- and subtype-independent manner could mitigate the impact of newly emergent virus strains when strain-matched vaccines are not yet available [5]–[7]. Universal vaccines would provide heterosubtypic immunity directed against viral components conserved among all influenza A viruses, and could be stockpiled for use in controlling outbreaks. While heterosubtypic immunity does not prevent infection, it can reduce morbidity and mortality and promote accelerated viral clearance [5], [6], resulting in a shortened period of viral shedding and reduced transmission of virus.

In recent years considerable attention has been paid to the challenge of generating effective immune responses at mucosal surfaces. Mucosal surfaces are sites of pathogen entry, replication and pathology, making immune responses at these locations critical for effective immunity [8]. Candidate influenza vaccines delivered intranasally and inducing mucosal immunity include live-attenuated viruses [9], adjuvanted killed vaccines [10], [11], and recombinant viral vectors. Recombinant adenovirus (rAd) vectors are potently immunogenic [12], [13] and induce effective pathogen-specific mucosal immunity when delivered intranasally [14]–[17]. rAd vectors are under investigation in more than 300 human gene therapy and vaccine clinical trials for a variety of indications [18], including several phase II and III studies (http://www.clinicaltrials.gov).

We and others have progressively refined DNA and rAd immunization strategies that induce heterosubtypic protection against influenza virus, including virulent H5N1 strains [19]–[22]. In a previous study, we demonstrated a prime-boost regimen that generated heterosubtypic immunity focused on conserved viral antigens and was able to protect both mice and ferrets from lethal influenza virus challenge [21]; others showed that this protection could be overcome with very high H5N1 challenge doses [23]. Crucially, prime-boost approaches require a protracted multi-dose immunization regimen, a drawback for use in response to emerging viruses. Building on this earlier work, we sought to streamline induction of heterosubtypic protection by harnessing the power of mucosal immunity.

Here we report a single-dose mucosal vaccination inducing heterosubtypic immunity using rAd vectors expressing the conserved viral antigens nucleoprotein (NP) and matrix 2 (M2). This strategy represents a strong candidate for a subtype-independent universal vaccine that could be stockpiled and deployed in the early stages of an influenza pandemic.

## Results

### Comparison of route of administration and antigen choice

**Figure 1:**
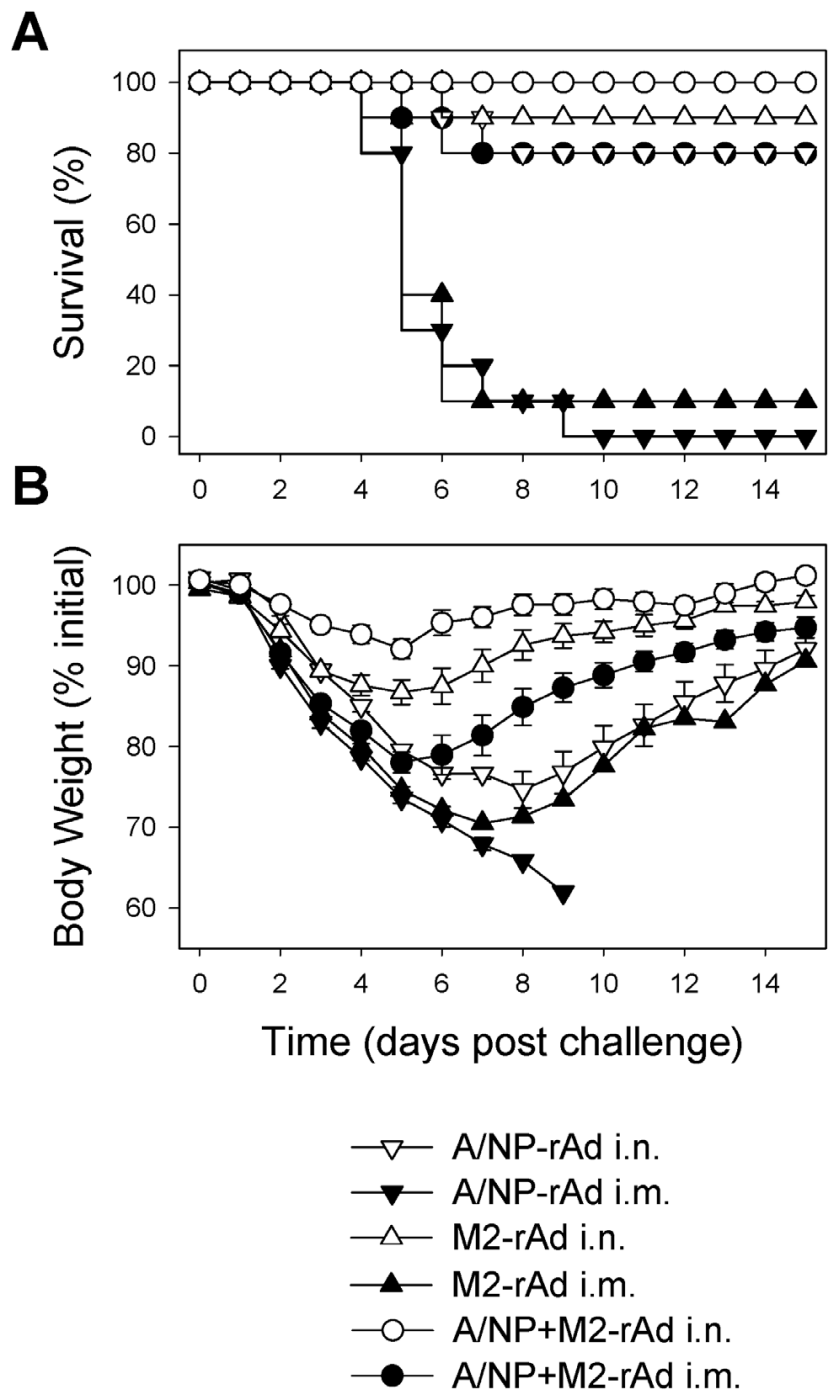
Single-dose i.n. rAd immunization protects from lethal influenza virus challenge. Groups of ten 12-week old BALB/cAnNCr mice were immunized with 1×10^10^ particles of A/NP-rAd or M2-rAd, or with 1×10^10^ particles each of A/NP and M2-rAd, via the i.m. or i.n. routes. 4 weeks after immunization, animals were challenged i.n. with 10^4^ TCID_50_ (100 LD_50_) A/FM/1/47-ma (H1N1) and monitored for survival (A) and weight loss (B).

Initial experiments compared effectiveness of a single intramuscular (i.m.) or intranasal (i.n.) immunization with A/NP-, M2-, or a combination of A/NP+M2-rAd against challenge infection with a virulent mouse-adapted H1N1 strain (A/FM). A/NP- or M2-rAd given i.m. failed to protect (defined by survival: see Materials and Methods) following A/FM challenge (**Figure 1**). The A/NP+M2-rAd combination given i.m. did protect, albeit with significant weight loss. In contrast, the same rAd-immunizations given i.n. were significantly more effective, with A/NP-rAd providing 80% survival (*P*<0.05 vs. i.m.), M2-rAd providing 90% survival (*P*<0.05 vs. i.m.), and the A/NP+M2-rAd combination providing complete protection. This enhanced protection was reflected in reduced weight loss. These results show that altering the route from parenteral injection to mucosal administration at an anatomically relevant site dramatically improves vaccine effectiveness. As A/NP+M2-rAd provided optimal protection, subsequent experiments focused on this combination.

### Intranasal rAd immunization triggers strong mucosal immune responses

Anatomically compartmentalized immune responses are observed in DNA prime-rAd boost immunization [21], so we explored this for single-dose rAd immunization. Mice were immunized i.m. or i.n. with A/NP+M2-rAd or rAd expressing influenza B virus nucleoprotein (B/NP-rAd) which serves as a specificity control to rule out innate immune protection due to the rAd vector. Antibody, T-cell, and cytokine responses were analyzed at one and ten months post-immunization.

**Figure 2:**
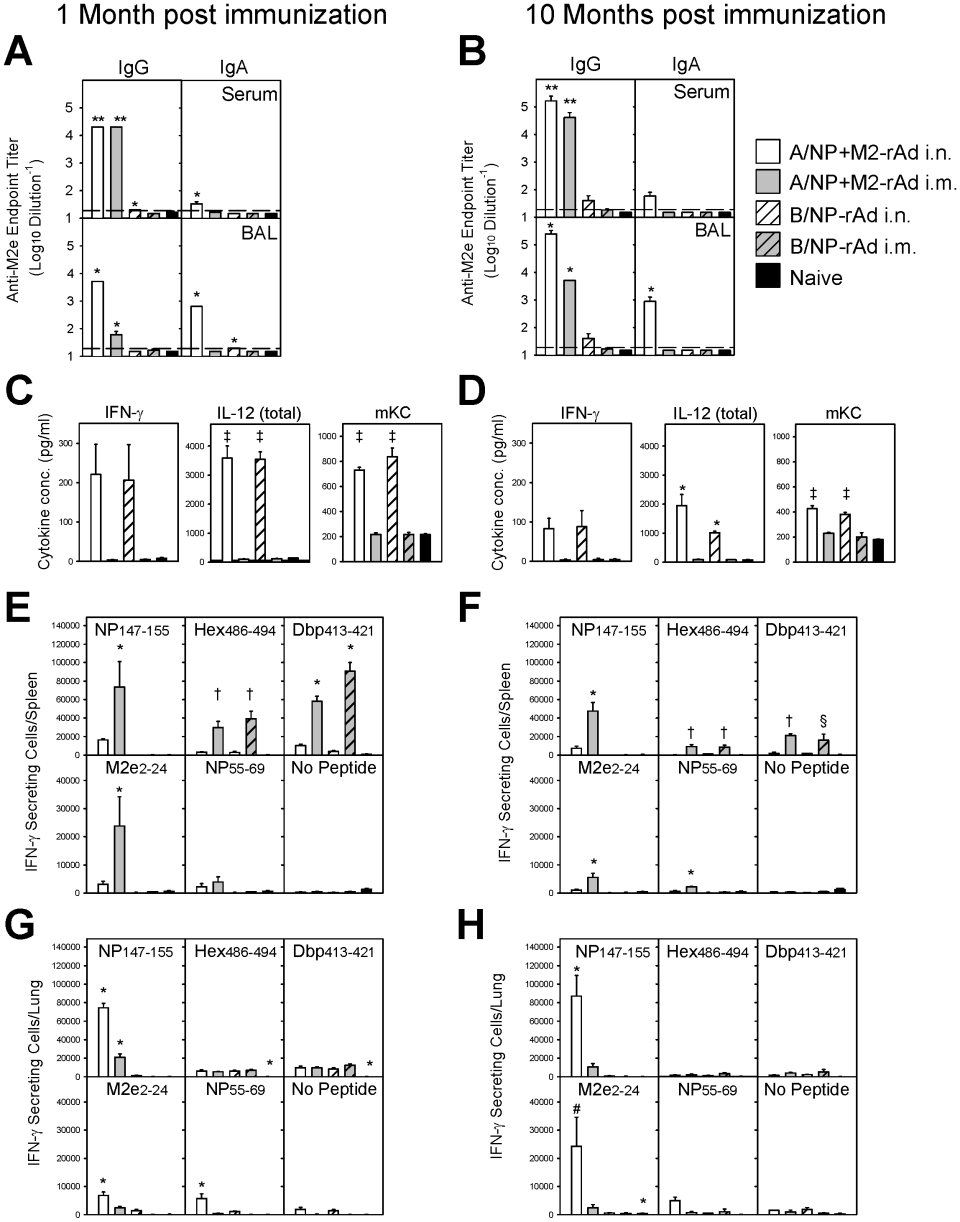
Immune responses after single-dose rAd immunization. BALB/cAnNCr mice were immunized with 5×10^9^ particles each of A/NP-rAd and M2-rAd, or 1×10^10^ particles of B/NP-rAd i.n. or i.m., or were unimmunized (naïve). Analyses were performed at one month (A, C, E, G) or 10 months (B, D, F, H) post-immunization. (A, B) M2e-specific IgG (left panels) and IgA (right panels) responses in serum and BAL were measured by ELISA as described. Bars show mean ± SEM of 3 mice per group. The dashed line indicates limit of detection. (C, D) Pro-inflammatory cytokine levels in BAL were measured as described. Bars show mean ± SEM of 4 mice per group at 1 month or 3 mice per group at 10 months. T-cell responses in spleen (E, F) and lung (G, H) were measured by IFN-γ ELISPOT of triplicate wells after stimulation with NP_147–155_, Hex_486–494_, Dbp_413–421_, M2e_2–24_, NP_55–69_ peptides. Unstimulated cells (no peptide) were used as controls. Bars show mean total IFN-γ secreting cell number per organ ± SEM of 4 mice per group at month or 3 mice per group at 10 months. Statistically significant differences are indicated as follows: * *P*<0.05 compared to all other groups; ‡ *P*<0.05 compared to i.m. and naïve groups; † *P*<0.05 compared to i.n. and naïve groups; ** *P*<0.05 compared to B/NP-rAd and naïve groups; § *P*<0.05 compared to A/NP+M2-rAd i.n. and naïve groups; # *P*<0.05 compared to all other groups except A/NP+M2-rAd i.m.

At one month post-A/NP+M2-rAd immunization, serum IgG levels against M2e were high and equivalent between mice vaccinated i.n. and i.m. (**Figure 2A**), but IgG levels in bronchoalveolar lavage (BAL) were higher after i.n. than i.m. immunization. IgA against M2e was detectable only following i.n. immunization, with levels in BAL considerably higher than in serum. These differences in the antibody response were maintained at 10 months post-immunization (**Figure 2B**). Similar results were seen for antibodies against rNP (**Figure S1**).

Elevated pro-inflammatory cytokine levels in BAL (IFN-γ, mKC, IL-12) were seen after i.n. but not i.m. rAd immunization, regardless of the transgene, at both one and 10 months post-immunization (**Figure 2C and D**). Levels of other cytokines in BAL (IL-1β, IL-2, IL-4, IL-5, IL-10 and TNF-α) were low at both one and 10 months and equivalent between groups (data not shown). Serum cytokine levels were low and similar between groups at both one and 10 months (data not shown).

T-cell responses against conserved viral epitopes were assessed by IFN-γ ELISPOT analysis of spleen (**Figure 2E and F**) and lung cells (**Figure 2G and H**). At one month post-immunization, strong responses to the immunodominant H2-K^d^ restricted NP_147–155_ epitope were seen in spleen after i.m. but not i.n. immunization with A/NP+M2-rAd. The converse was observed in lungs, with approximately 3-fold more NP_147–155_-specific lung cells per mouse following i.n. compared to i.m. immunization. Reponses to MHC-II restricted epitopes (M2e_2–24_ and NP_55–69_) followed a similar pattern but were of lower magnitude. Responses to rAd epitopes Hex_486–494_ and Dbp_413–421_ were independent of the transgene and much higher in spleen after i.m. than after i.n. immunization, but were similar in magnitude in lungs regardless of immunization route. Similar compartmentalization of the T-cell response was observed at 10 months post-immunization. Responses to the dominant NP147_–_155 epitope in both spleen and lungs were similar in magnitude to those at 1 month. In contrast, the M2e2-24-specific response in the spleen was lower than at 1 month but was considerably greater in the lungs of A/NP+M2-rAd i.n. mice. The response to rAd epitopes was much lower at 10 months than at one month regardless of the route of administration or transgene.

**Figure 3:**
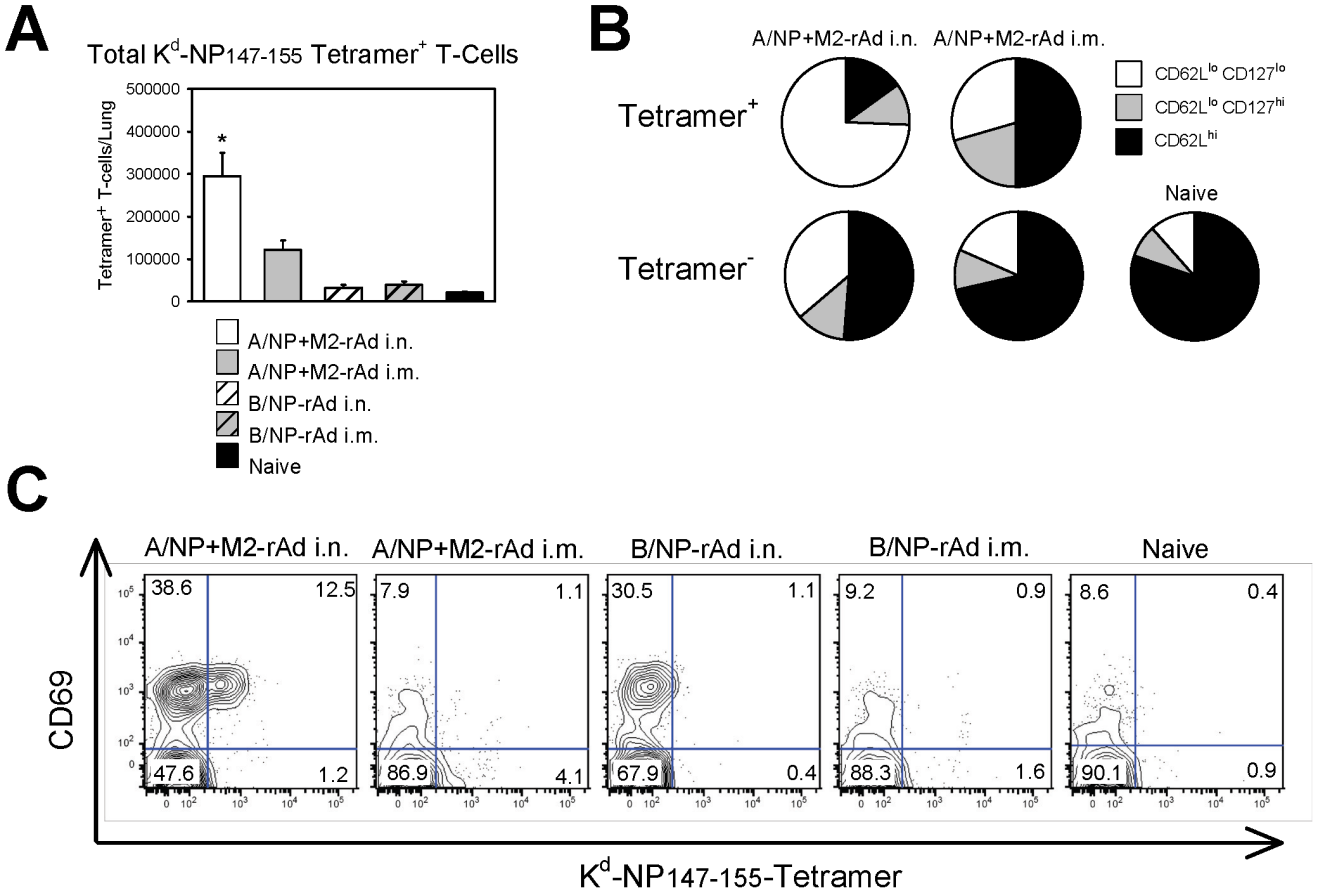
Lung T cell phenotyping. BALB/cAnNCr mice were immunized with 5×10^9^ particles each of A/NP-rAd and M2-rAd, or 1×10^10^ particles of B/NP-rAd i.n. or i.m., or were unimmunized (naïve). Lung T cells were isolated and analyzed by flow cytometry. (A) Total number of K^d^-NP_147–155_-tetramer positive CD8^+^ T cells recovered from the lungs of mice, as determined by multicolor flow cytometry. Bars show mean ± SEM of 3 animals per group. (B) Phenotypic analysis of lung CD8^+^ T cells. Pie charts show relative proportions of central memory (CD62L^hi^), effector memory (CD62L^lo^, CD127^hi^) and activated effector memory (CD62L^lo^, CD127^lo^) among tetramer positive (upper pies) and tetramer negative (lower pies) CD8^+^ T-cells. (C) Activation status of lung CD8^+^ T cells, as determined by staining for tetramer vs. CD69. Each plot shows one representative mouse per group of 3 mice assessed. Numbers in plots indicate % of CD8^+^ T cells per quadrant. * Indicates a statistically significant difference (*P*<0.05) compared to all other groups.

Tetramer staining confirmed that animals immunized with A/NP+M2-rAd i.n. had more NP_147–155_-specific CD8^+^ T cells recoverable from lungs than i.m. immunized animals (**Figure 3A**). Compared to i.m. rAd immunized mice, a greater proportion of tetramer positive cells from lungs of i.n. rAd immunized mice showed an effector memory (CD62L^lo^) phenotype, and the majority were also CD127^lo^ (**Figure 3B**). A smaller proportion of cells with this activated phenotype were seen in the NP_147–155_-tetramer negative population, which contains T cells specific to other epitopes including the rAd vector. More CD8^+^ T cells (both tetramer positive and negative) expressed the early activation marker CD69 after i.n. than i.m. immunization (**Figure 3C**), indicating that not only did i.n. rAd immunization result in greater virus-specific CD8^+^ T-cell responses in the lung, but these cells also possessed a more activated phenotype than cells induced by i.m. immunization.

### Intranasal rAd immunization provides long-lived protection from virulent influenza virus challenge

**Figure 4:**
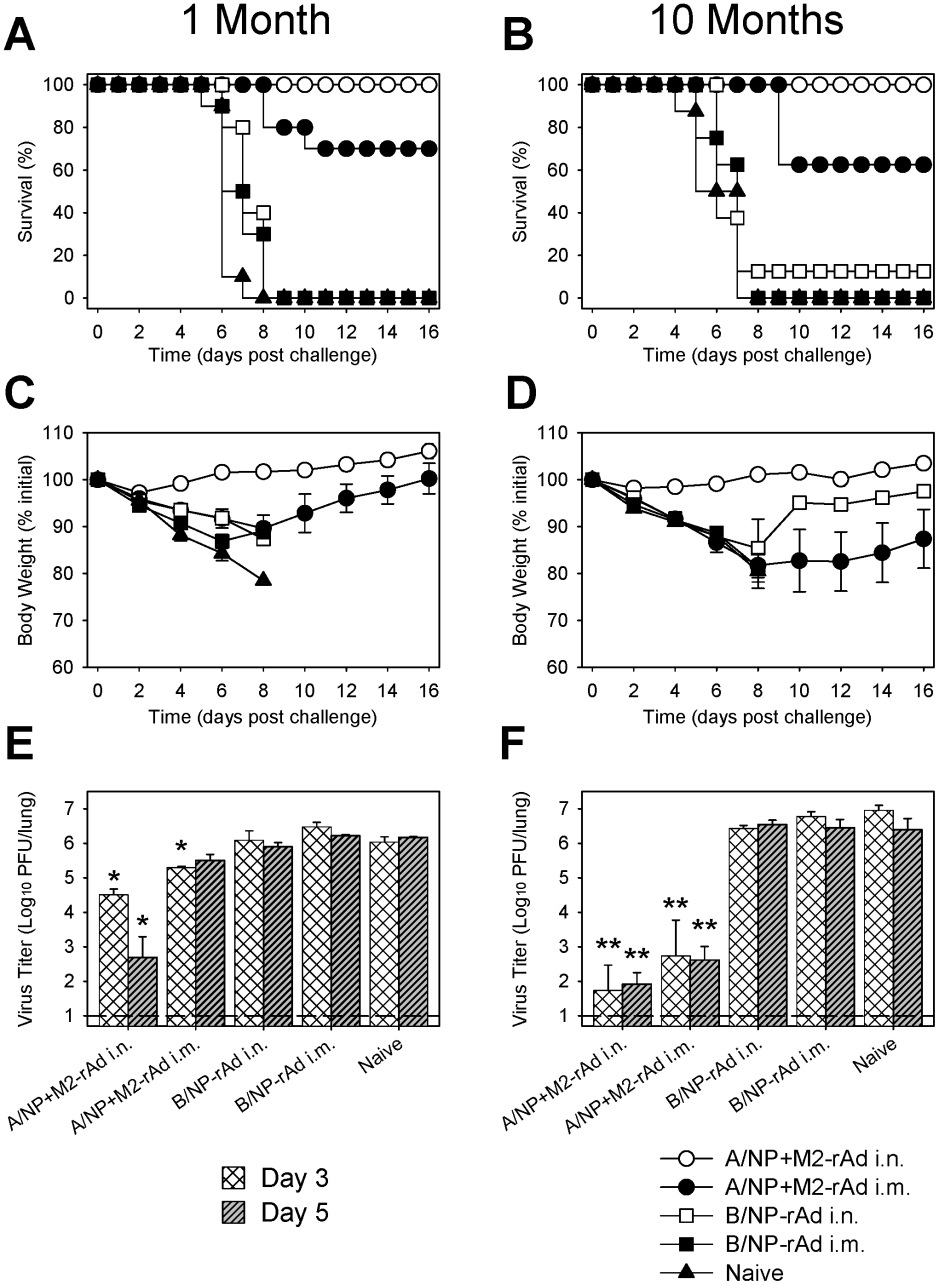
Morbidity and mortality after H5N1 challenge following single-dose rAd immunization. Groups of 10 (at one month) or 8 (at 10 months) BALB/cAnNCr mice were challenged with 10^2.84^ EID_50_ (∼10 LD_50_) of A/VN1203 one month (A, B, C) or 10 months (D, E, F) after immunization. (A, D) Survival after challenge. (B, E) Weight loss after challenge. When challenged one month post-boosting, statistically significant differences in weight loss (*P*<0.05) were observed between A/NP+M2-rAd i.n. and all other groups at days 4–12. When challenged at 10 months, weight loss in A/NP+M2-rAd i.n. immunized mice was significantly (*P*<0.05) different from all other groups from days 4–16. (C, F) Virus titers in the lungs at days 3 and 5 after challenge as determined by plaque assay. Bars show log_10_ geometric mean titer ± SEM of 4 mice per group. The dashed line shows limit of detection. * indicates a statistically significant difference (*P*<0.05) compared to all other groups at the same time point; ** indicates a significant difference from B/NP-rAd and naïve groups.

Immunized animals were tested for protection against challenge with a highly pathogenic H5N1 strain with pandemic potential (A/VN1203) at both one and 10 months post-immunization. At one month after immunization, all animals in the A/NP+M2-rAd i.n. group were protected and had little weight loss (**Figure 4**). In contrast, the A/NP+M2-rAd i.m. group suffered significant weight loss and 30% died by day 11. Control animals died by day 8. Lung virus titers 3 days post-challenge were significantly lower in mice immunized with A/NP+M2-rAd by either route than in controls, with titers significantly lower after A/NP+M2-rAd i.n. than i.m. immunization. At day 5, only A/NP+M2-rAd i.n. immunized mice had significantly lower titers than controls. When challenged 10 months after immunization, control animals died with comparable kinetics to those challenged at 1 month. All A/NP+M2-rAd i.n. immunized animals were protected with little weight loss, but A/NP+M2-rAd i.m. immunized animals suffered more severe weight loss than at 1 month and 37.5% succumbed to infection (**Figure 4D and E**). Lung virus titers at both days 3 and 5 post-challenge were significantly lower than controls in mice immunized with A/NP+M2-rAd via either route. Interestingly, virus titers in A/NP+M2-rAd immunized mice were lower at 10 months than at one month (**Figure 4C and F**). Despite these reduced lung virus titers, the A/NP+M2-rAd i.m. group had worse outcomes than at one month. The reasons for this are unclear.

Protection was also assessed in immunized animals challenged with a virulent mouse-adapted H3N2 strain [X-79] (**Figure S2**). Complete protection was seen in mice immunized with A/NP+M2-rAd via either route when challenged with X-79 one month after vaccination, although significantly greater weight loss (*P*<0.05) was observed for i.m. than i.n. immunized animals. Lung virus titers at day 3 were lower in the A/NP+M2-rAd groups than in control groups, with titers significantly lower (*P*<0.05) after i.n. compared to i.m. immunization. At day 5, lung virus titers were further reduced and similar between A/NP+M2-rAd i.m. and i.n. groups. At 10 months post-A/NP+M2-rAd immunization, complete protection with minimal weight loss was maintained in mice immunized i.n., but was waning in animals immunized i.m. (75% protection with severe weight loss). This is reflected in day 3 lung virus titers, which were significantly lower in A/NP+M2-rAd i.n. mice than in all other groups.

### IgA is not required for protection

**Figure 5:**
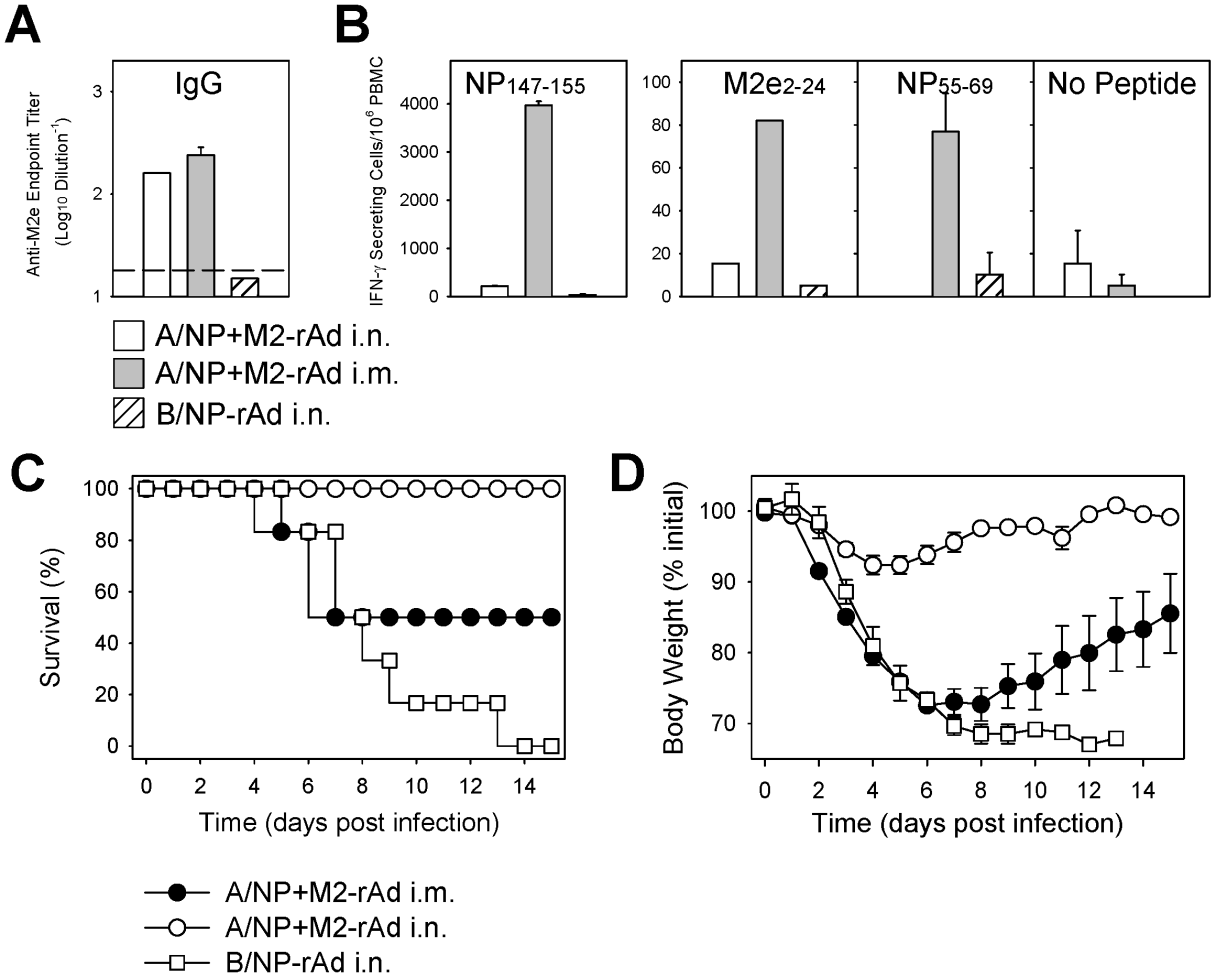
IgA is not required for protection after i.n. rAd immunization. Groups of 6 IgA^−/−^-BALB/c mice were immunized with 5×10^9^ particles each of A/NP-rAd and M2-rAd i.n. or i.m., or 1×10^10^ particles of B/NP-rAd i.n. (A) IgG responses against M2e were measured by ELISA using serum obtained 2 weeks after immunization. The dashed line indicates limit of detection. (B) Antigen-specific T-cell responses at 3 weeks post-immunization were determined in peripheral blood pooled from these animals by IFN-γ ELISPOT using NP_147–155_, NP_55–69_ or M2e_2-24_ peptides as stimulus. Unstimulated cells (no peptide) were used as a control. Bars show mean ± SEM of triplicate wells for each group per stimulus. At one month post-immunization, animals were challenged with 10^4^ TCID_50_ (100 LD_50_) of A/FM and monitored for survival (C) and weight loss (D). Error bars in weight loss graph indicate mean ± SEM. Statistically significant differences in weight loss (*P*<0.05) were observed between A/NP+M2-rAd i.n. and i.m. at days 2–15, between A/NP+M2-rAd i.n. and B/NP-rAd at days 3–13, and between A/NP+M2-rAd i.n. and B/NP-rAd at days 2, 3, 9 and 13.

As virus-specific IgA was detectable after i.n. but not i.m. rAd immunization, the role of this response in enhanced protection was examined. BALB/c-IgA^−/−^ mice were immunized with A/NP+M2-rAd i.n. or i.m., or with B/NP-rAd i.n. as a control. M2e-specific IgG was detectable in serum 2 weeks post-immunization (**Figure 5A**) and the IFN-γ ELISPOT response in PBMCs (**Figure 5B**) was similar to wild-type mice (**see Figure S3B**). When challenged with A/FM one month post-immunization all control animals died, while i.m. immunized animals lost considerable weight and 50% died (**Figure 5C and D**). The i.n. immunized animals lost significantly less weight than i.m. immunized mice and survived challenge, indicating that while IgA may play a role following i.n. rAd immunization, it is not required for protection and is likely not the sole reason for superiority of mucosal immunization.

### Intranasal immunization rapidly induces protection

**Figure 6:**
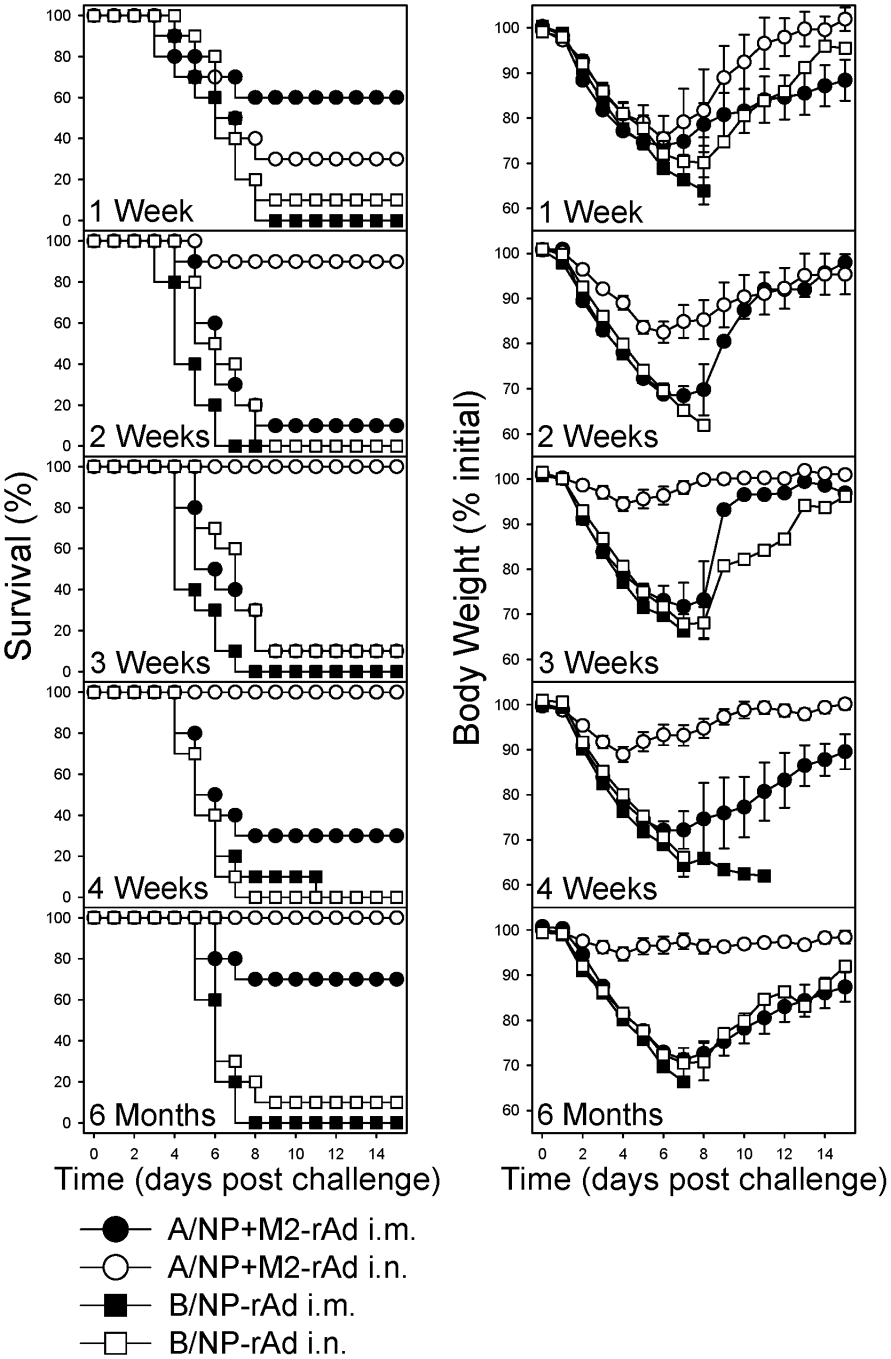
Kinetics of protection after single-dose rAd immunization. BALB/cAnNCr mice were immunized with 5×10^9^ particles each of A/NP-rAd and M2-rAd, or 1×10^10^ particles of B/NP-rAd i.n. or i.m. and challenged with 10^4^ TCID_50_ (∼100 LD_50_) of A/FM at 1, 2, 3, or 4 weeks or 6 months later. Left panels show survival and right panels show weight loss after challenge. Groups consisted of 10 mice per immunization per challenge time. Error bars in weight loss graphs indicate mean ± SEM. Statistically significant (*P*<0.05) differences in survival were as follows: A/NP+M2-rAd i.n. was significantly different from A/NP+M2-rAd i.m. at weeks 2, 3, and 4, and from B/NP-rAd i.n. or i.m. at all times; A/NP+M2-rAd i.m. was significantly different from B/NP-rAd i.m. at 2 weeks and 6 months, but not different from B/NP-rAd i.n. at any time. In terms of weight loss, no statistically significant differences were seen between groups at week 1. At week 2 A/NP+M2-rAd i.n. differed (*P*<0.05) from all other groups on days 2–7, and also from B/NP-rAd i.n. on day 1; A/NP+M2-rAd i.m. was significantly different from B/NP-rAd i.n. on days 2–3 and from B/NP-rAd i.m. on day 1. At week 3 A/NP+M2-rAd differed from all other groups on days 2–8; B/NP-rAd i.n. differed from all other groups on day 3. At week 4 A/NP+M2-rAd i.n. was significantly different from all other groups on days 2–15; A/NP+M2-rAd i.m. differed from B/NP-rAd i.m. on day 11. At 6 months A/NP+M2-rAd i.n. was significantly different from all other groups on days 2–10, and from A/NP+M2-rAd i.m. on days 11–15.

Universal vaccines could be deployed when strain-matched vaccines are unavailable, providing interim protection early in a pandemic. To evaluate this potential, we examined how rapidly protection develops. Mice were immunized i.n. or i.m., then challenged with A/FM at various times post-immunization and monitored for survival and weight loss (**Figure 6**). Control animals immunized with B/NP-rAd lost significant weight and died. Partial protection was observed in A/NP+M2-rAd i.m. mice challenged one week post-immunization although animals lost significant weight. This partial protection was lost at 2 and 3 weeks but began to reappear at 4 weeks and good protection was observed at 6 months despite significant weight loss. Animals immunized with A/NP+M2-rAd i.n. showed little protection at one week but had 90% protection at 2 weeks and complete protection with little weight loss from 3 weeks out to at least 6 months post-immunization.

Examination of antibody levels in the serum revealed that IgG specific to M2e was detectable within one week of i.m. immunization and within 2 weeks for i.n. immunization (**Figure S3A**). IgG reached maximal levels by 5 weeks and remained high for at least 6 months. Serum IgA responses became detectable in i.n. immunized mice by 2 weeks after immunization for M2e. The IgA response was absent in the case of i.m. immunization. Again, development of a virus-specific serum IgA response correlated with protection for the i.n. rAd immunized groups. However there was no serum antibody correlate for the partial protection observed in i.m. immunized mice at one week; although IgG responses against M2e were observed early after i.m. immunization, they increased further at weeks 2–5 as protection decreased.

As assessed by IFN-γ ELISPOT, virus specific T cell levels in peripheral blood were modest one week after i.m. rAd immunization, higher at 2–4 weeks, and then declined to approximately one third of peak levels at 6 months post-immunization (**Figure S3B**). As expected, strong responses were seen against the immunodominant NP147_–_155 peptide, with lower responses directed to NP_55–69_ and M2e_2–24_ helper T cell epitopes. Peripheral blood T-cell responses to NP_147–155_ following i.n. rAd immunization were lower and rose later than after i.m. immunization but did not decline by 6 months (**Figure S3B**).

### Intranasal rAd immunization confers accelerated challenge virus clearance

**Figure 7:**
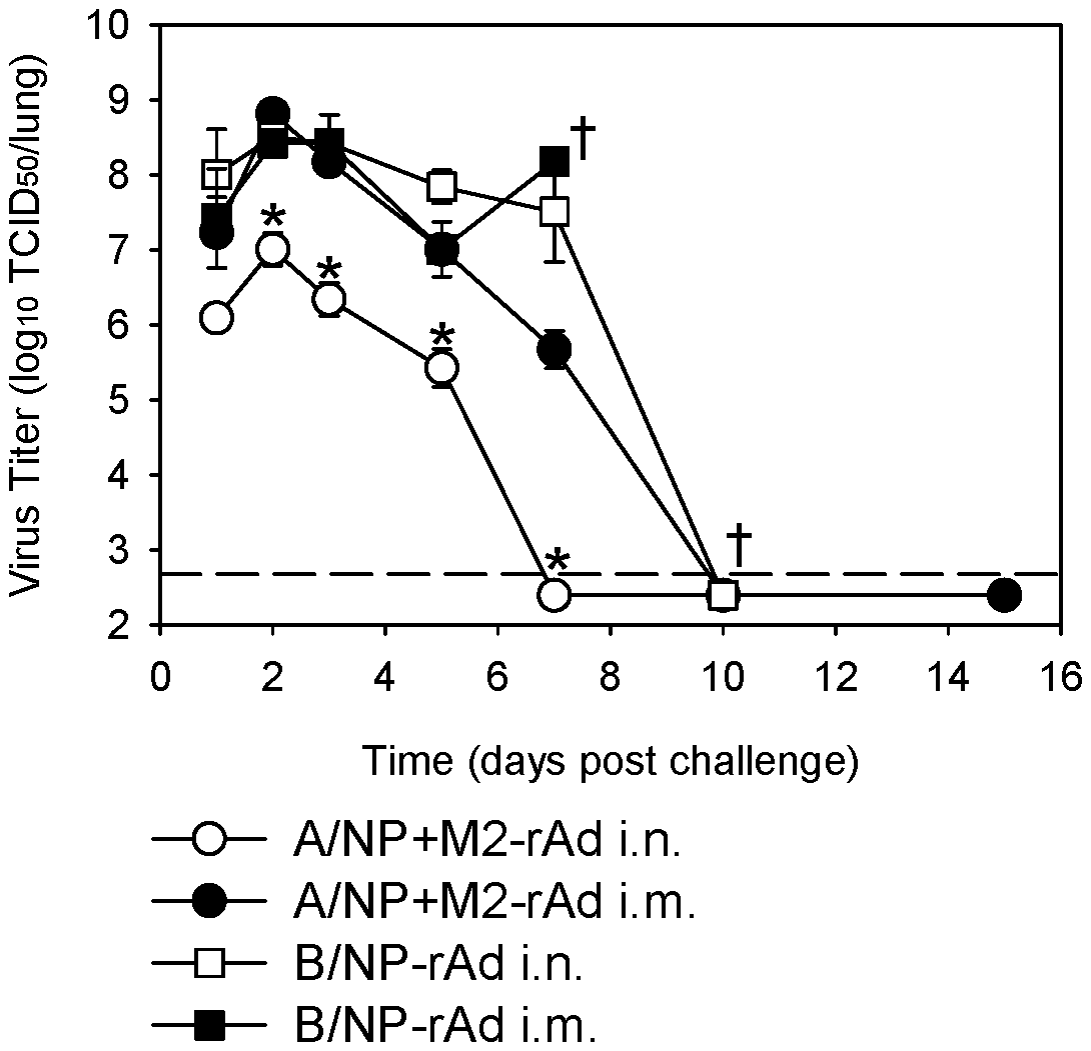
Kinetics of virus clearance following H1N1 challenge. BALB/cAnNCr mice were immunized with 5×10^9^ particles each of A/NP-rAd and M2-rAd, or 1×10^10^ particles of B/NP-rAd i.n. or i.m. and challenged with 10^4^ TCID_50_ (∼100 LD_50_) of A/FM one month later. Lung virus titers were measured by TCID_50_ at the indicated time points, and are expressed as geometric mean titer ± SEM of 3 mice per group per time point, except for day 7 in the B/NP-rAd i.m. group and day 10 in the B/NP-rAd i.n. group where only 1 mouse remained alive (indicated by †). The dashed line shows limit of detection. * indicates a statistically significant difference (*P*<0.001) compared to all other groups at the same time point.

Kinetic studies of lung virus titers were performed to assess rate of virus clearance after A/FM challenge. Maximal titers were seen 2 days post-challenge in all groups (**Figure 7**) with B/NP-rAd immunized mice retaining high titers through day 7. A/NP+M2-rAd i.m. immunized mice had peak virus titers similar to those of B/NP-rAd mice but exhibited a steady decrease in titer from day 3 onward, with virus clearance complete by day 10. Mice immunized with A/NP+M2-rAd i.n. showed significantly lower titers (*P*<0.001) as early as day 2 post-challenge and cleared virus more rapidly than the other groups, with clearance complete by day 7.

## Discussion

The recent emergence of an unanticipated pandemic virus has highlighted the need for influenza vaccines providing broad coverage across multiple strains and subtypes. This study demonstrates that a single dose of rAd vaccines expressing two highly conserved influenza virus antigens (NP and M2) protects from virulent influenza virus challenges with multiple widely divergent subtypes. Protection is greatly enhanced by i.n. administration, develops by 2 weeks post-immunization, and persists for at least 10 months. Intranasal rAd vaccination decreased lung virus titers and accelerated challenge virus clearance relative to i.m. vaccination. This reduction in virus titers corresponded to greatly reduced morbidity, as shown by weight loss, compared to controls and i.m. immunized animals.

Antibody and T-cell responses against both NP and M2 were seen. Previous work suggests that dominant protective mechanisms are likely M2e-specific antibodies that limit viral spread [24] and mediate antibody-dependent cellular cytotoxicity [25], and NP-specific CD8^+^ T cells eliminating infected cells by cytolysis [26]. M2e and NP-specific helper T cells probably make a lesser contribution as direct anti-viral effectors. A role for NP-specific antibody has also been proposed [27], but the significance of this in protection is debated. As with prime-boost studies [20], [21], the A/NP+M2 rAd vector combination was superior to either component alone in providing protection, which may be due to induction of complementary effector mechanisms including virus-specific IgA and T cells in the respiratory tract.

Targeting multiple viral antigens, in this case NP and M2, clearly has advantages over using a single antigen. Immunization with either M2-rAd or A/NP-rAd alone confers less protection than using a mixture of these two rAd vectors (**Figure 1**). The A/NP+M2-rAd combination also has potential advantages in preventing emergence of viral escape mutants, which have been reported for NP [28], [29] and M2 [30]. However, even under selective pressure from monoclonal antibodies, very few escape mutant sequences were observed for M2e [30] suggesting that this region is biologically constrained. Combination vaccines also reduce the possibility that individuals with certain HLA types may be overall non-responders to the vaccine. The latter is a potential problem with M2 due to the small size of this protein (97 amino acids) and the limited number of potential epitopes it may contain, but less of a concern with the larger NP (498 amino acids) within which multiple epitopes for numerous MHC-I and –II alleles have been identified [31], [32]. It is possible that incorporation of additional antigens (such as M1 or PB1) into heterosubtypic vaccines may further broaden and enhance the response.

HA-expressing rAd influenza vaccines intended to induce strain-matched neutralizing antibodies have been studied in mice [33], [34], chickens [35], and humans [36], but would require regular reformulation to accommodate antigenic variation in HA. Recent studies show that monoclonal antibodies against conserved epitopes in the HA stem region can neutralize viruses of several subtypes [37]–[39]. However, such antibodies do not react with all HA subtypes, so multiple such immunogens would be needed to cover all subtypes. NP-expressing rAd5 and chimp rAd vectors given i.m. provided partial protection from both H1N1 and H5N1 challenges in mice [40], but required a higher vaccine dose than reported here. Our findings demonstrate that a single dose of the A/NP+M2-rAd combination given i.n. provided complete protection against highly virulent H1N1, H3N2, and H5N1 influenza virus challenges.

Compared to the DNA prime-rAd boost approach previously reported [21], the single-dose rAd strategy reported here has the advantage of a streamlined vaccination protocol. Significantly, it obviates the requirement for multiple vaccine doses used in prime-boost regimens (3 doses of DNA given at 2 week intervals by i.m. injection, followed by rAd given i.m. or i.n. one month later is typical). We have previously demonstrated that for NP-based immunization, DNA priming enhances protection over that of i.m. rAd alone [41]. However, results reported here suggest that priming is less critical when rAd is given intranasally.

The public health applications of prime-boost and single-dose rAd vaccination are quite different. DNA priming could be administered during routine health care to establish basal immunity. It would offer partial protection in the event of an unexpected outbreak or drift, as well as priming for enhanced responses to rAd or other viral boosts. The DNA vaccines can be given repeatedly without concern about anti-vector immunity. In contrast, the single dose rAd vaccination could be used on an urgent basis early in a pandemic or unexpected outbreak.

Single-dose i.n. rAd protected within 2 weeks, with maximal protection by 3 weeks, which compares favorably with strain-matched vaccines. Immunity induced by single-dose rAd is also long-lived, with complete protection observed for at least 10 months after i.n. immunization. Although i.m. rAd provided some protection, this did not prevent weight loss and was waning at 10 months. Durable protection is important for vaccines designed to cover the gap between emergence of a new strain and the availability of matched vaccines.

Concern has been raised about the possibility of adenovirus vectors accessing the central nervous system. Although transgene expression in the olfactory bulb occurs after i.n. administration of rAd vectors to mice, this is transient, low level, and not associated with inflammation [42], [43]. Another potential limitation of rAd vaccines is host immunity to the vector which may interfere with vaccination [12]. This is a particular concern for Ad5-based vectors, to which much of the human population has neutralizing antibody [44]. While experimental animals mount strong immune responses against rAd vectors, this is not always the case in humans. In gene therapy trials some individuals do not make neutralizing antibody responses against rAd given i.n., even after repeated administrations [45]. Other reports suggest that rAd immunization may induce *de novo* T-cell responses against the transgene despite pre-existing immunity, albeit lower levels than in seronegative individuals [46]. To circumvent immunity, vectors based on rare adenovirus serotypes [47], non-human primate adenoviruses [48], or chimeric viruses [49] have been suggested in place of current Ad5 vectors.

The rAd doses used here are broadly similar to those used in other vaccine studies. It may be possible to use lower rAd doses. Preliminary results using A/NP-rAd given i.n. suggest that similar levels of protection are achieved with a 10-fold lower rAd dose (**Figure S4**). Studies to assess minimum protective doses for the A/NP+M2-rAd combination are ongoing. An effective rAd vaccine dose for humans would have to be addressed in future clinical trials, and may not require a dose proportionate to body weight. Further vector optimization (for example by encoding both NP and M2 on a bicistronic vector) may be possible, and could allow vaccine dose to be reduced still further. It should be noted that since rAd-based vaccines targeting conserved antigens would not need to be changed frequently, vaccine manufacture could occur on an ongoing basis to produce a stockpile, rather than on a seasonal basis as is the case for current influenza vaccines.

Immune correlates of protection are needed for new vaccine types. IgA is not required for protection, but may play a role in protection when present, and could provide a useful correlate. Serum IgG responses do not correlate with protection. They are similar between i.n. and i.m. rAd immunizations which differ in protection (**Figure 2A and B**; **Figure S1**), and develop earlier after i.m. than i.n. rAd, strengthening at 2 weeks as protection decreases (**Figure S3A**). Mucosal IgG appears more promising, as i.n. rAd immunization induced higher BAL IgG responses than i.m. rAd. Interestingly, this indicates that i.n. rAd immunization likely induces IgG-secreting cells resident within the respiratory tract; if antibody reached the BAL by transudation from serum (where IgG levels are similar between i.n. and i.m. immunized mice), then BAL IgG levels would be equivalent regardless of immunization route.

Ideally, correlates of protection should be feasible to assess in humans with non-invasive sampling methods. Anatomical compartmentalization of cellular immune populations after i.n. immunization (ref. 21 and this study) complicates the matter. Cellular correlates can be identified, for example IFN-γ secreting virus-specific T cells in the lungs, but cannot be directly measured in humans. However, the frequency of IFN-γ secreting cells in blood increases between 1 and 6 months after i.n. immunization (**Figure S3B**), suggesting equilibration between lung and blood T cell pools over time. If the relevant lung T cells possess a distinctive phenotype of memory, homing or activation markers, perhaps low numbers of comparable cells could be detected in the circulation.

Our studies using a non-replicating viral vector rather than productive infection are in agreement with reports that virus-specific T cells resident in the lungs after clearance of viral infection exhibit an activated phenotype in both mice [50], [51] and humans [52]. While the classic paradigm is that during recall responses memory T cells activated in draining lymph nodes recirculate back to the site of infection to clear pathogen, T cells already present in tissue and re-activated locally may be able to mediate immediate effector function to control virus [5]. This agrees with our observation that virus titers were significantly reduced from 2 days post-challenge, with a trend for lower titers at day 1, in i.n. but not i.m. immunized animals.

Detection of elevated cytokine levels (IFN-γ, mKC, IL-12) in BAL at both one and 10 months after i.n. rAd vaccination (**Figure 2C and D**) is surprising. This was transgene-independent, and thus due to the rAd vector. mKC (CXCL1) is a powerful neutrophil chemoattractant and functional homolog of human IL-8/CXCL8 [53], [54]. IFN-γ is immunostimulatory and secreted by various cells including activated T cells and macrophages [55]. The elevated IFN-γ levels in BAL after i.n. rAd immunization could result from continued IL-12 secretion. IL-12 promotes differentiation of CD4^+^ T cells towards a Th1 phenotype [56] and maintains CD4^+^ IFN-γ^+^ T cell effector function [57]. Continued IL-12 expression in BAL after i.n. rAd immunization may maintain CD4^+^ T cell activation, which could sustain the strong virus-specific CD8^+^ T-cell responses observed in the lung.

We have not yet identified the cellular source of the cytokines seen in BAL, but rAd can infect immature dendritic cells (DC) from both mice and humans, causing them to mature and secrete IL-12 [58], [59]. This occurs independently of transgene expression [58] via a TLR9/MyD88 dependent pathway *in vitro* [60]. Earlier studies demonstrated that exposure to an aerosolized antigen induced an activated CD11c^+^ CD11b^+^ DC subset in BAL that retained antigen presenting function for several weeks after antigen exposure [61].

Other studies have been interpreted as showing persistence of influenza virus antigen after infection, and have suggested that this maintains the activation of virus-specific T cells generated in response to infection [51], [62]–[64]. In contrast to the situation with natural influenza virus infection, previous studies have demonstrated persistence of both vector genome and antigen expression for at least a year following i.m. rAd immunization of mice [65]. This sustained antigen expression appears important for supporting the activated phenotype of transgene-specific T cells following rAd immunization [65], [66], although maintenance of memory T cell populations eventually becomes antigen-independent [66]. Differences in expression pattern likely explain the dichotomy between the T-cell responses against the transgenes (NP and M2 which are driven by a constitutively active CMV promoter) which were sustained over the duration of the study and responses against the rAd vector (the E1, E3 deleted vector backbone driving only minimal and transient expression of Hexon and DNA-binding protein) which decline substantially over this time. Ultimately, the combination of prolonged low-level antigen presentation and Th1 cytokine production may be optimal for maintaining protective mucosal immune responses.

With their ability to induce potent innate and adaptive immune responses and to deliver antigen to intracellular processing and presentation pathways, rAd vectors may be particularly well suited for vaccination against viruses and other intracellular pathogens. Here we demonstrate their potential as an emergency, fast-acting vaccine inducing long-lasting protective immunity in the respiratory tract. Heterosubtypic rAd vaccines could be stockpiled in advance since regular reformulation would be unnecessary, and could be delivered by nasal spray, facilitating widespread administration by limited healthcare personnel. During a large scale virus outbreak or pandemic, reducing disease severity by vaccination, even while allowing mild infection, could greatly reduce the burden on healthcare facilities. Conserved antigen vaccines do not provide the type of sterilizing immunity mediated by HA-specific neutralizing antibody, but would permit only a mild, transient natural influenza virus infection. This transient infection would further boost heterosubtypic immunity and induce neutralizing antibodies against the exact virus strain circulating in the community, preventing re-infection.

## Materials and Methods

### Ethics statement

All animal protocols and procedures were approved by Institutional Animal Care and Use Committees at the Center for Biologics Evaluation and Research (CBER; Protocol #1991-06) and/or the Centers for Disease Control and Prevention (CDC; Protocol #1619) in animal facilities accredited by the Association for Assessment and Accreditation of Laboratory Animal Care International. All experiments were performed according to institutional guidelines.

### rAd vaccines

Recombinant adenovirus vectors (Ad5-ΔE1ΔE3) expressing A/NP, B/NP or consensus M2 have been described previously [22], [41]. Briefly, Pac I-linearized A/NP- or B/NP-containing shuttle vectors were recombined with a cosmid containing Ad5 genomic DNA using Cre-recombinase. M2-rAd was constructed using the ViraPower Adenoviral Expression System (Invitrogen, Carlsbad, CA) by subcloning the M2-consensus sequence into the pAd/CMV/V5-DEST Gateway vector using LR Clonase. In each case, recombinants were transfected into 293 cells for recovery of rAd vectors. High titer rAd stocks were prepared by ViraQuest Inc. (North Liberty, IA) and stored at −80°C in PBS with 3% sucrose. Viral particle concentration was determined by absorbance at 260 nm. All rAd stocks were confirmed negative for replication-competent adenovirus by passage on non-permissive cells.

### Influenza viruses

The highly virulent, mouse-adapted virus A/FM/1/47-ma (H1N1) [A/FM] [67] originally provided by Earl Brown (University of Ottawa, Canada), the mouse-adapted A/Philippines/2/82 X A/PR/8/34 (H3N2) reassortant [X-79] [68], and the systemically replicating avian strain A/Vietnam/1203/2004 (H5N1) [A/VN1203] [69] have been described previously. Virus stocks were prepared as previously described [20]. Experiments involving H5N1 virus were conducted under enhanced BSL-3 containment at CDC; all other experiments were conducted under BSL-2 conditions at CBER.

### Experimental animals, immunization, and challenge infection

Female BALB/cAnNCr mice aged 8–12 weeks purchased from the National Cancer Institute, Frederick, MD were used for all experiments, except where indicated. IgA^−/−^ mice were originally obtained by MTA from Baylor University [70] and backcrossed onto the BALB/c background in our colony using marker-assisted accelerated backcrossing (MAX-BAX) technology from Charles River Laboratories (Wilmington, MA). BALB/c-IgA^−/−^ mice were bred in the CBER colony. Intramuscular injection of rAd vaccine used a 100 µl volume split evenly between each rear quadriceps muscle; intranasal immunization used a 50 µl volume delivered under isoflurane anesthesia. H1N1 and H3N2 challenge infections were performed under isoflurane anesthesia; 2,2,2-tribromoethanol in *tert*-amyl alcohol (Avertin; Aldrich Chemical Co., Milwaukee, WI) given i.p. was used as anesthetic for H5N1 challenge. Following challenge infection, animals were monitored for weight loss and survival. In this paper the term “protection” is defined as survival following challenge infection, and does not imply complete absence of virus replication.

### Mucosal sampling

Mice were euthanized with ketamine/xylazine, and bronchoalveolar lavage (BAL) and lung cells were obtained as described previously [21].

### Peptides and proteins

The following peptides were synthesized by the CBER core facility: NP_147–155_ (TYQRTRALV), NP_55–69_ (RLIQNSLTIERMVLS), and M2e_2–24_ consensus sequence (SLLTEVETPIRNEWGCRCNDSSD) corresponding to the surface exposed M2 ectodomain region (M2e). The MHC-I restricted adenovirus 5 hexon (Hex_486–494_: KYSPSNVKI) and DNA-binding protein (Dbp_419–427_: FALSNAEDL) peptides [71] were synthesized by GenScript (Piscataway, NJ). Recombinant nucleoprotein (rNP) from strain A/PR/8/34 (H1N1) was purchased from Imgenex (San Diego, CA).

### T cell ELISPOT

Interferon (IFN)- γ ELISPOT was performed as previously described [22] on lung and spleen cells by stimulation with indicated peptides.

### Flow cytometry

Lung and spleen T cell phenotypes were assessed by surface staining with CD3-eFluor450, CD8-APC-eFluor780, CD62L-PE-Cy7, CD69-PE, CD127-PerCP-Cy5.5 (all from eBiosciences, San Diego, CA), NP_147–155_-H2-K^d^ Tetramer-APC (NIH Tetramer core facility, Atlanta, GA), and Live/Dead fixable viability stain for 488 nm excitation (Invitrogen, Carlsbad, CA). 50,000 events were acquired on a BD-FACS Canto II and data analyzed with FlowJo (TreeStar, Ashland, OR) software. Thresholds of positivity were identified using fluorescence minus one controls for each color on cells from each sample group.

### Cytokine Analysis

Cytokine levels in BAL were assessed using the Meso Scale Discovery mouse Th1/Th2 9-plex ultra-sensitive kit (MSD, Gaithersburg, MD), according to manufacturer's instructions. This kit allows simultaneous quantitation of murine IL-1β, IL-2, IL-4, IL-5, IL-10, total IL-12, IFN-γ, mKC, and TNF-α.

### Antibody analysis

Antibody levels in serum and BAL were assessed by ELISA, as described previously [20], [21]. Data are expressed as endpoint titers, defined as the highest dilution of sample giving an OD 405 nm reading greater than 3 SD above the mean of the naïve samples.

### Virus titration

Virus titers in tissues were determined by plaque assay (for A/VN1203) or TCID_50_ (for A/FM) as described previously [21], [72].

### Statistical analysis

SigmaStat v 3.5 (Systat Software, Point Richmond, CA) was used for all statistical analyses. Body weight and virus titers analyses used one way ANOVA, followed by pairwise multiple comparisons via the Holm-Sidak method. Survival analysis was performed by the Log-Rank method.

## Supporting Information

Figure S1 Serum and BAL antibody responses to nucleoprotein. Mice were immunized with 5×10^9^ particles each of A/NP-rAd and M2-rAd, or 1×10^10^ particles of B/NP-rAd i.n. or i.m., or were unimmunized (naïve). rNP-specific IgG (left panels) and IgA (right panels) responses in serum and BAL were measured by ELISA as described one month (A) and 10 months (B) after immunization. Bars show mean ± SEM of 3 mice per group. The dashed line indicates limit of detection. Statistically significant differences are indicated as follows: * *P*<0.05 compared to all other groups; ** *P*<0.05 compared to B/NP-rAd and naïve groups.
(0.56 MB TIF)

Figure S2 Morbidity and mortality after H3N2 challenge following single dose rAd immunization. Groups of 10 (at one month) or 8 (at 10 months) mice were challenged with 5×10^4^ TCID_50_ (∼100 LD_50_) of X-79 one month (A, B and C) or 10 months (D, E and F) after immunization. (A, D) Survival after challenge. (B, E) Weight loss after challenge. When challenged one month post boosting statistically significant differences in weight loss (*P*<0.05) were observed between A/NP+M2-rAd i.n. and i.m. groups at days 1–7, between A/NP+M2-rAd i.n. and B/NP-rAd or naïve groups at days 1–15, and between A/NP+M2-rAd and B/NP or naive groups at days 5–15. When challenged at 10 months weight loss in A/NP+M2-rAd i.n. immunized mice was significantly (*P*<0.05) different from all other groups from days 3–11 and 13–15. (C, F) Virus titers in the lungs at days 3 and 5 after challenge, as determined by TCID_50_. Bars show log_10_ geometric mean titer ± SEM of 4 mice per group, or 3 mice per group at 10 months. The dashed line shows limit of detection. * indicates a statistically significant difference (*P*<0.05) compared to all other groups at the same time point; ** indicates a significant difference from B/NP-rAd and naïve groups; (dagger} indicates a significant difference from B/NP-rAd groups. Note that day 5 titers were not assessed at 10 months.
(0.68 MB TIF)

Figure S3 Kinetics of the immune response after single-dose rAd immunization. Mice were immunized with 5×10^9^ particles each of A/NP-rAd and M2-rAd, or 1×10^10^ particles of B/NP-rAd i.n. or i.m. (A) M2e-specific IgG (left panels) and IgA (right panels) responses in serum collected 1, 2, 3, or 5 weeks or 6 months post-immunization were measured by ELISA. Bars show endpoint titer of serum pooled from 10 mice per group. The dashed line indicates limit of detection. For measurement of T-cell responses by IFN-γ ELISPOT (B), peripheral blood from 10 mice per group was collected and pooled at 1, 2, 3, or 4 weeks or 6 months post-immunization, as indicated. T-cell responses were determined using NP_147–155_, NP_55–69_ or M2e_2–24_ peptides as stimulus. Unstimulated cells (no peptide) were used as a control. Bars show mean ± SEM of triplicate wells for each group per stimulus. For the A/NP+M2-rAd i.m. group at 2 weeks, specific T-cell responses could not be determined due to high background observed in all test wells. This is indicated by HB in (B). This high background was not seen in a repeat experiment.
(0.63 MB TIF)

Figure S4 Dose titration of i.n. A/NP-rAd immunization. Groups of 12-week old BALB/cAnNCr mice were immunized i.n. with 1×10^10^ (10 mice), 1×10^9^ (5 mice), or 1×10^8^ (5 mice) particles of A/NP-rAd. 4 weeks after immunization, animals were challenged i.n. with 10^4^ TCID_50_ (100 LD_50_) A/FM/1/47-ma (H1N1) and monitored for survival (A) and weight loss (B).
(0.57 MB TIF)

## References

[1] Carrat F, Flahault A (2007). Influenza vaccine: the challenge of antigenic drift.. Vaccine.

[2] Leroux-Roels I, Leroux-Roels G (2009). Current status and progress of prepandemic and pandemic influenza vaccine development.. Expert Rev Vaccines.

[3] ‘Interim results: influenza A (H1N1) 2009 monovalent vaccination coverage --- United States, October-December 2009.’. MMWR Morb Mortal Wkly Rep.

[4] Dawood FS, Jain S, Finelli L, Shaw MW, Lindstrom S (2009). Emergence of a novel swine-origin influenza A (H1N1) virus in humans.. N Engl J Med.

[5] Brown LE, Kelso A (2009). Prospects for an influenza vaccine that induces cross-protective cytotoxic T lymphocytes.. Immunol Cell Biol.

[6] Epstein SL (2003). Control of influenza virus infection by immunity to conserved viral features.. Expert Rev Anti Infect Ther.

[7] Schotsaert M, De FM, Fiers W, Saelens X (2009). Universal M2 ectodomain-based influenza A vaccines: preclinical and clinical developments.. Expert Rev Vaccines.

[8] Belyakov IM, Ahlers JD (2009). What role does the route of immunization play in the generation of protective immunity against mucosal pathogens?. J Immunol.

[9] Murphy BR, Coelingh K (2002). Principles underlying the development and use of live attenuated cold-adapted influenza A and B virus vaccines.. Viral Immunol.

[10] Takada A, Matsushita S, Ninomiya A, Kawaoka Y, Kida H (2003). Intranasal immunization with formalin-inactivated virus vaccine induces a broad spectrum of heterosubtypic immunity against influenza A virus infection in mice.. Vaccine.

[11] Tumpey TM, Renshaw M, Clements JD, Katz JM (2001). Mucosal delivery of inactivated influenza vaccine induces B-cell-dependent heterosubtypic cross-protection against lethal influenza A H5N1 virus infection.. J Virol.

[12] Barouch DH, Nabel GJ (2005). Adenovirus vector-based vaccines for human immunodeficiency virus type 1.. Hum Gene Ther.

[13] Draper SJ, Heeney JL (2010). Viruses as vaccine vectors for infectious diseases and cancer.. Nat Rev Microbiol.

[14] Wang J, Thorson L, Stokes RW, Santosuosso M, Huygen K (2004). Single mucosal, but not parenteral, immunization with recombinant adenoviral-based vaccine provides potent protection from pulmonary tuberculosis.. J Immunol.

[15] Xiang ZQ, Yang Y, Wilson JM, Ertl HC (1996). A replication-defective human adenovirus recombinant serves as a highly efficacious vaccine carrier.. Virology.

[16] Xu Q, Pichichero ME, Simpson LL, Elias M, Smith LA (2009). An adenoviral vector-based mucosal vaccine is effective in protection against botulism.. Gene Ther.

[17] Yu JR, Kim S, Lee JB, Chang J (2008). Single intranasal immunization with recombinant adenovirus-based vaccine induces protective immunity against respiratory syncytial virus infection.. J Virol.

[18] Edelstein ML, Abedi MR, Wixon J (2007). Gene therapy clinical trials worldwide to 2007–an update.. J Gene Med.

[19] Lalor PA, Webby RJ, Morrow J, Rusalov D, Kaslow DC (2008). Plasmid DNA-based vaccines protect mice and ferrets against lethal challenge with A/Vietnam/1203/04 (H5N1) influenza virus.. J Infect Dis.

[20] Lo CY, Wu Z, Misplon JA, Price GE, Pappas C (2008). Comparison of vaccines for induction of heterosubtypic immunity to influenza A virus: cold-adapted vaccine versus DNA prime-adenovirus boost strategies.. Vaccine.

[21] Price GE, Soboleski MR, Lo CY, Misplon JA, Pappas C (2009). Vaccination focusing immunity on conserved antigens protects mice and ferrets against virulent H1N1 and H5N1 influenza A viruses.. Vaccine.

[22] Tompkins SM, Zhao ZS, Lo CY, Misplon JA, Liu T (2007). Matrix protein 2 vaccination and protection against influenza viruses, including subtype H5N1.. Emerg Infect Dis.

[23] Rao SS, Kong WP, Wei CJ, Van HN, Gorres JP (2010). Comparative efficacy of hemagglutinin, nucleoprotein, and matrix 2 protein gene-based vaccination against H5N1 influenza in mouse and ferret.. PLoS One.

[24] Zebedee SL, Lamb RA (1988). Influenza A virus M2 protein: monoclonal antibody restriction of virus growth and detection of M2 in virions.. J Virol.

[25] Jegerlehner A, Tissot A, Lechner F, Sebbel P, Erdmann I (2002). A molecular assembly system that renders antigens of choice highly repetitive for induction of protective B cell responses.. Vaccine.

[26] Karzon DT (1996). Cytotoxic T cells in influenza immunity.. Semin Virol.

[27] Carragher DM, Kaminski DA, Moquin A, Hartson L, Randall TD (2008). A novel role for non-neutralizing antibodies against nucleoprotein in facilitating resistance to influenza virus.. J Immunol.

[28] Boon AC, de Mutsert G, Graus YM, Fouchier RA, Sintnicolaas K (2002). Sequence variation in a newly identified HLA-B35-restricted epitope in the influenza A virus nucleoprotein associated with escape from cytotoxic T lymphocytes.. J Virol.

[29] Voeten JT, Bestebroer TM, Nieuwkoop NJ, Fouchier RA, Osterhaus AD (2000). Antigenic drift in the influenza A virus (H3N2) nucleoprotein and escape from recognition by cytotoxic T lymphocytes.. J Virol.

[30] Zharikova D, Mozdzanowska K, Feng J, Zhang M, Gerhard W (2005). Influenza type A virus escape mutants emerge in vivo in the presence of antibodies to the ectodomain of matrix protein 2.. J Virol.

[31] Bui HH, Peters B, Assarsson E, Mbawuike I, Sette A (2007). Ab and T cell epitopes of influenza A virus, knowledge and opportunities.. Proc Natl Acad Sci U S A.

[32] Heiny AT, Miotto O, Srinivasan KN, Khan AM, Zhang GL (2007). Evolutionarily conserved protein sequences of influenza A viruses, avian and human, as vaccine targets.. PLoS One.

[33] Hoelscher MA, Garg S, Bangari DS, Belser JA, Lu X (2006). Development of adenoviral-vector-based pandemic influenza vaccine against antigenically distinct human H5N1 strains in mice.. Lancet.

[34] Holman DH, Wang D, Raja NU, Luo M, Moore KM (2008). Multi-antigen vaccines based on complex adenovirus vectors induce protective immune responses against H5N1 avian influenza viruses.. Vaccine.

[35] Gao W, Soloff AC, Lu X, Montecalvo A, Nguyen DC (2006). Protection of mice and poultry from lethal H5N1 avian influenza virus through adenovirus-based immunization.. J Virol.

[36] Van Kampen KR, Shi Z, Gao P, Zhang J, Foster KW (2005). Safety and immunogenicity of adenovirus-vectored nasal and epicutaneous influenza vaccines in humans.. Vaccine.

[37] Ekiert DC, Bhabha G, Elsliger MA, Friesen RH, Jongeneelen M (2009). Antibody recognition of a highly conserved influenza virus epitope.. Science.

[38] Okuno Y, Isegawa Y, Sasao F, Ueda S (1993). A common neutralizing epitope conserved between the hemagglutinins of influenza A virus H1 and H2 strains.. J Virol.

[39] Sui J, Hwang WC, Perez S, Wei G, Aird D (2009). Structural and functional bases for broad-spectrum neutralization of avian and human influenza A viruses.. Nat Struct Mol Biol.

[40] Roy S, Kobinger GP, Lin J, Figueredo J, Calcedo R (2007). Partial protection against H5N1 influenza in mice with a single dose of a chimpanzee adenovirus vector expressing nucleoprotein.. Vaccine.

[41] Epstein SL, Kong WP, Misplon JA, Lo CY, Tumpey TM (2005). Protection against multiple influenza A subtypes by vaccination with highly conserved nucleoprotein.. Vaccine.

[42] Damjanovic D, Zhang X, Mu J, Fe MM, Xing Z (2008). Organ distribution of transgene expression following intranasal mucosal delivery of recombinant replication-defective adenovirus gene transfer vector.. Genet Vaccines Ther.

[43] Lemiale F, Kong WP, Akyurek LM, Ling X, Huang Y (2003). Enhanced mucosal immunoglobulin A response of intranasal adenoviral vector human immunodeficiency virus vaccine and localization in the central nervous system.. J Virol.

[44] Tatsis N, Ertl HC (2004). Adenoviruses as vaccine vectors.. Mol Ther.

[45] Harvey BG, Hackett NR, El-Sawy T, Rosengart TK, Hirschowitz EA (1999). Variability of human systemic humoral immune responses to adenovirus gene transfer vectors administered to different organs.. J Virol.

[46] Catanzaro AT, Koup RA, Roederer M, Bailer RT, Enama ME (2006). Phase 1 safety and immunogenicity evaluation of a multiclade HIV-1 candidate vaccine delivered by a replication-defective recombinant adenovirus vector.. J Infect Dis.

[47] Thorner AR, Lemckert AA, Goudsmit J, Lynch DM, Ewald BA (2006). Immunogenicity of heterologous recombinant adenovirus prime-boost vaccine regimens is enhanced by circumventing vector cross-reactivity.. J Virol.

[48] Tatsis N, Tesema L, Robinson ER, Giles-Davis W, McCoy K (2006). Chimpanzee-origin adenovirus vectors as vaccine carriers.. Gene Ther.

[49] Roberts DM, Nanda A, Havenga MJ, Abbink P, Lynch DM (2006). Hexon-chimaeric adenovirus serotype 5 vectors circumvent pre-existing anti-vector immunity.. Nature.

[50] Hogan RJ, Usherwood EJ, Zhong W, Roberts AD, Dutton RW (2001). Activated antigen-specific CD8+ T cells persist in the lungs following recovery from respiratory virus infections.. J Immunol.

[51] Zammit DJ, Turner DL, Klonowski KD, Lefrancois L, Cauley LS (2006). Residual antigen presentation after influenza virus infection affects CD8 T cell activation and migration.. Immunity.

[52] de Bree GJ, van Leeuwen EM, Out TA, Jansen HM, Jonkers RE (2005). Selective accumulation of differentiated CD8+ T cells specific for respiratory viruses in the human lung.. J Exp Med.

[53] Bozic CR, Kolakowski LF, Gerard NP, Garcia-Rodriguez C, von Uexkull-Guldenband C (1995). Expression and biologic characterization of the murine chemokine KC.. J Immunol.

[54] Zlotnik A, Yoshie O (2000). Chemokines: a new classification system and their role in immunity.. Immunity.

[55] Young HA, Hardy KJ (1995). Role of interferon-gamma in immune cell regulation.. J Leukoc Biol.

[56] Szabo SJ, Sullivan BM, Peng SL, Glimcher LH (2003). Molecular mechanisms regulating Th1 immune responses.. Annu Rev Immunol.

[57] Stobie L, Gurunathan S, Prussin C, Sacks DL, Glaichenhaus N (2000). The role of antigen and IL-12 in sustaining Th1 memory cells in vivo: IL-12 is required to maintain memory/effector Th1 cells sufficient to mediate protection to an infectious parasite challenge.. Proc Natl Acad Sci U S A.

[58] Zhang Y, Chirmule N, Gao GP, Qian R, Croyle M (2001). Acute cytokine response to systemic adenoviral vectors in mice is mediated by dendritic cells and macrophages.. Mol Ther.

[59] Zhong L, Granelli-Piperno A, Choi Y, Steinman RM (1999). Recombinant adenovirus is an efficient and non-perturbing genetic vector for human dendritic cells.. Eur J Immunol.

[60] Yamaguchi T, Kawabata K, Koizumi N, Sakurai F, Nakashima K (2007). Role of MyD88 and TLR9 in the innate immune response elicited by serotype 5 adenoviral vectors.. Hum Gene Ther.

[61] Julia V, Hessel EM, Malherbe L, Glaichenhaus N, O'Garra A (2002). A restricted subset of dendritic cells captures airborne antigens and remains able to activate specific T cells long after antigen exposure.. Immunity.

[62] Jelley-Gibbs DM, Brown DM, Dibble JP, Haynes L, Eaton SM (2005). Unexpected prolonged presentation of influenza antigens promotes CD4 T cell memory generation.. J Exp Med.

[63] Jelley-Gibbs DM, Dibble JP, Brown DM, Strutt TM, McKinstry KK (2007). Persistent depots of influenza antigen fail to induce a cytotoxic CD8 T cell response.. J Immunol.

[64] Kim TS, Hufford MM, Sun J, Fu YX, Braciale TJ (2010). Antigen persistence and the control of local T cell memory by migrant respiratory dendritic cells after acute virus infection.. J Exp Med.

[65] Tatsis N, Fitzgerald JC, Reyes-Sandoval A, Harris-McCoy KC, Hensley SE (2007). Adenoviral vectors persist in vivo and maintain activated CD8+ T cells: implications for their use as vaccines.. Blood.

[66] Finn JD, Bassett J, Millar JB, Grinshtein N, Yang TC (2009). Persistence of transgene expression influences CD8+ T-cell expansion and maintenance following immunization with recombinant adenovirus.. J Virol.

[67] Smeenk CA, Brown EG (1994). The influenza virus variant A/FM/1/47-MA possesses single amino acid replacements in the hemagglutinin, controlling virulence, and in the matrix protein, controlling virulence as well as growth.. J Virol.

[68] Chen KS, Quinnan GV (1988). Induction, persistence and strain specificity of haemagglutinin-specific secretory antibodies in lungs of mice after intragastric administration of inactivated influenza virus vaccines.. J Gen Virol.

[69] Maines TR, Lu XH, Erb SM, Edwards L, Guarner J (2005). Avian influenza (H5N1) viruses isolated from humans in Asia in 2004 exhibit increased virulence in mammals.. J Virol.

[70] Harriman GR, Bogue M, Rogers P, Finegold M, Pacheco S (1999). Targeted deletion of the IgA constant region in mice leads to IgA deficiency with alterations in expression of other Ig isotypes.. J Immunol.

[71] McKelvey T, Tang A, Bett AJ, Casimiro DR, Chastain M (2004). T-cell response to adenovirus hexon and DNA-binding protein in mice.. Gene Ther.

[72] Zeng H, Goldsmith C, Thawatsupha P, Chittaganpitch M, Waicharoen S (2007). Highly pathogenic avian influenza H5N1 viruses elicit an attenuated type I interferon response in polarized human bronchial epithelial cells.. J Virol.

