# Supplementary figures and images for: Single-Dose Mucosal Immunization with a Candidate Universal Influenza Vaccine Provides Rapid Protection from Virulent H5N1, H3N2 and H1N1 Viruses
Source: PLoS One. 2010 Oct 4;5(10):e13162. doi: 10.1371/journal.pone.0013162 (PMC2953831; doi:10.1371/journal.pone.0013162)

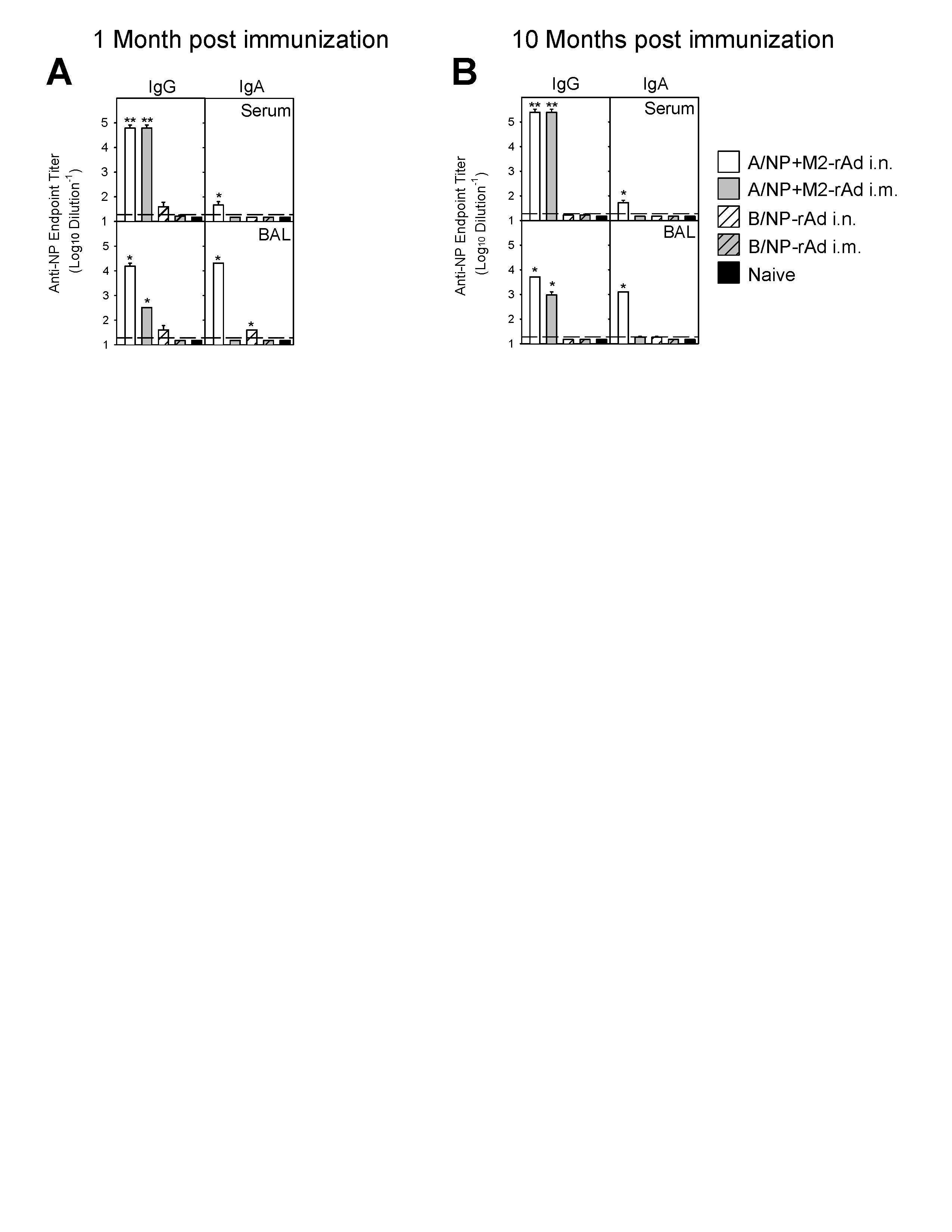

Supplement: Figure S1 — Serum and BAL antibody responses to nucleoprotein. Mice were immunized with 5×10^9^ particles each of A/NP-rAd and M2-rAd, or 1×10^10^ particles of B/NP-rAd i.n. or i.m., or were unimmunized (naïve). rNP-specific IgG (left panels) and IgA (right panels) responses in serum and BAL were measured by ELISA as described one month (A) and 10 months (B) after immunization. Bars show mean ± SEM of 3 mice per group. The dashed line indicates limit of detection. Statistically significant differences are indicated as follows: * P<0.05 compared to all other groups; ** P<0.05 compared to B/NP-rAd and naïve groups. (0.56 MB TIF) [file pone.0013162.s001.tif]

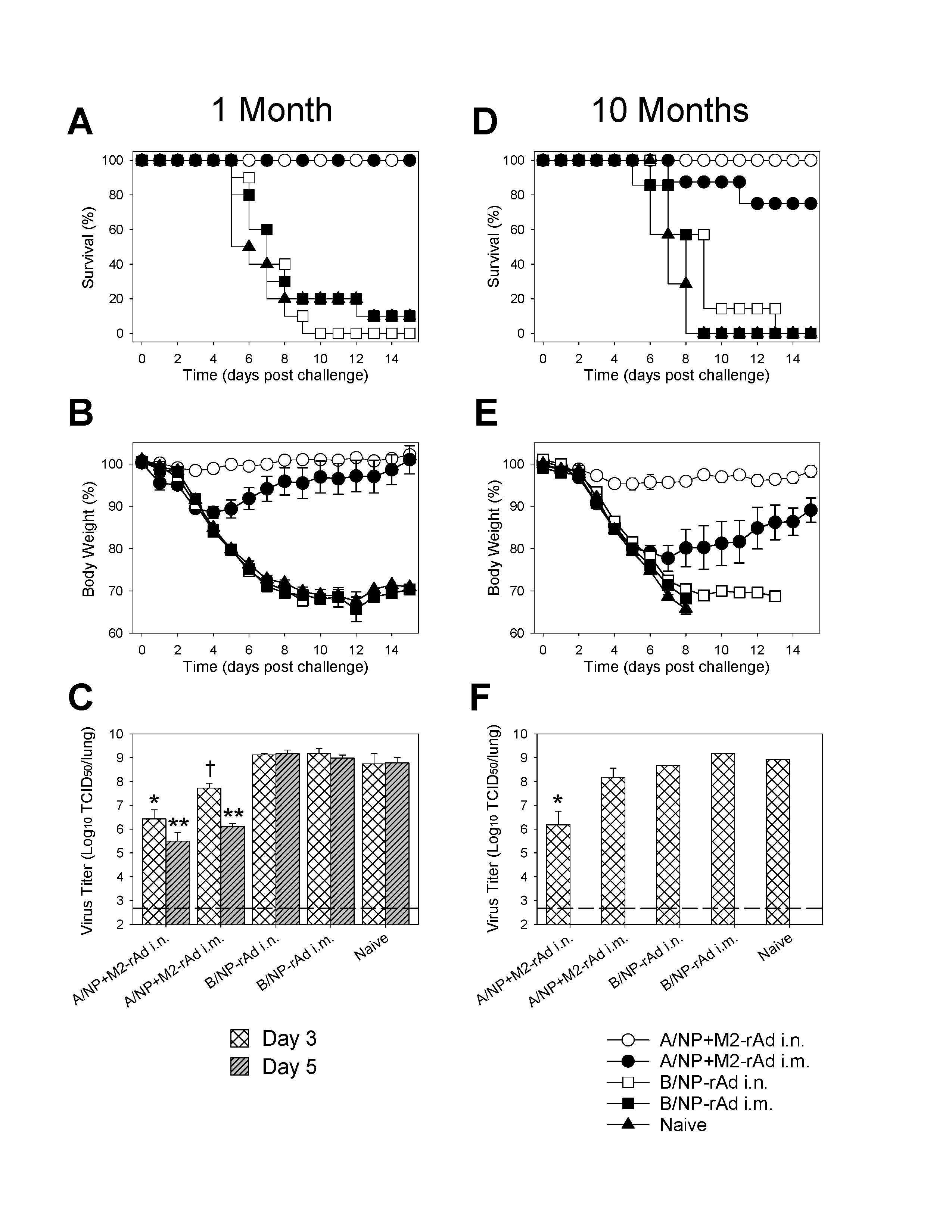

Supplement: Figure S2 — Morbidity and mortality after H3N2 challenge following single dose rAd immunization. Groups of 10 (at one month) or 8 (at 10 months) mice were challenged with 5×10^4^ TCID50 (∼100 LD50) of X-79 one month (A, B and C) or 10 months (D, E and F) after immunization. (A, D) Survival after challenge. (B, E) Weight loss after challenge. When challenged one month post boosting statistically significant differences in weight loss (P<0.05) were observed between A/NP+M2-rAd i.n. and i.m. groups at days 1–7, between A/NP+M2-rAd i.n. and B/NP-rAd or naïve groups at days 1–15, and between A/NP+M2-rAd and B/NP or naive groups at days 5–15. When challenged at 10 months weight loss in A/NP+M2-rAd i.n. immunized mice was significantly (P<0.05) different from all other groups from days 3–11 and 13–15. (C, F) Virus titers in the lungs at days 3 and 5 after challenge, as determined by TCID50. Bars show log10 geometric mean titer ± SEM of 4 mice per group, or 3 mice per group at 10 months. The dashed line shows limit of detection. * indicates a statistically significant difference (P<0.05) compared to all other groups at the same time point; ** indicates a significant difference from B/NP-rAd and naïve groups; (dagger} indicates a significant difference from B/NP-rAd groups. Note that day 5 titers were not assessed at 10 months. (0.68 MB TIF) [file pone.0013162.s002.tif]

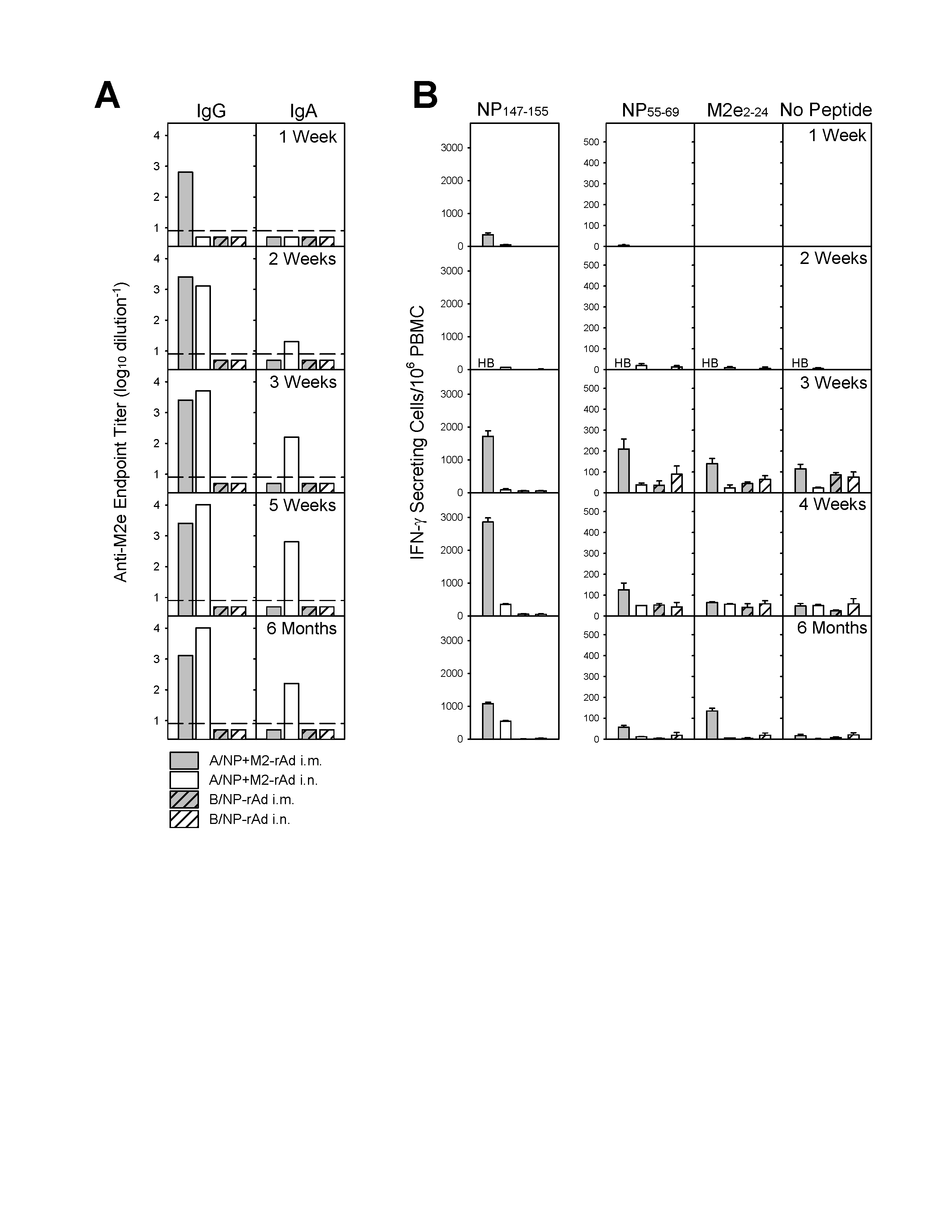

Supplement: Figure S3 — Kinetics of the immune response after single-dose rAd immunization. Mice were immunized with 5×10^9^ particles each of A/NP-rAd and M2-rAd, or 1×10^10^ particles of B/NP-rAd i.n. or i.m. (A) M2e-specific IgG (left panels) and IgA (right panels) responses in serum collected 1, 2, 3, or 5 weeks or 6 months post-immunization were measured by ELISA. Bars show endpoint titer of serum pooled from 10 mice per group. The dashed line indicates limit of detection. For measurement of T-cell responses by IFN-γ ELISPOT (B), peripheral blood from 10 mice per group was collected and pooled at 1, 2, 3, or 4 weeks or 6 months post-immunization, as indicated. T-cell responses were determined using NP147–155, NP55–69 or M2e2–24 peptides as stimulus. Unstimulated cells (no peptide) were used as a control. Bars show mean ± SEM of triplicate wells for each group per stimulus. For the A/NP+M2-rAd i.m. group at 2 weeks, specific T-cell responses could not be determined due to high background observed in all test wells. This is indicated by HB in (B). This high background was not seen in a repeat experiment. (0.63 MB TIF) [file pone.0013162.s003.tif]

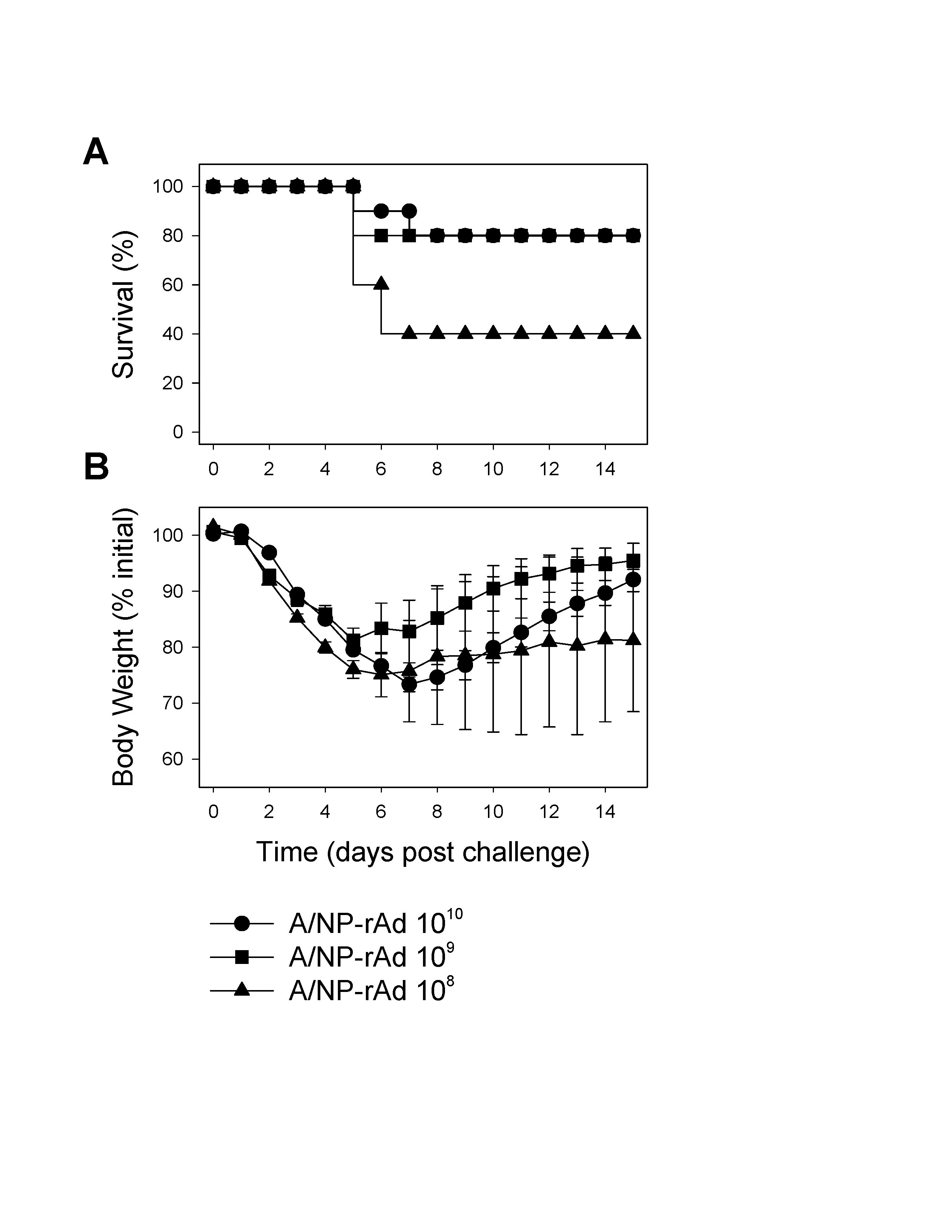

Supplement: Figure S4 — Dose titration of i.n. A/NP-rAd immunization. Groups of 12-week old BALB/cAnNCr mice were immunized i.n. with 1×10^10^ (10 mice), 1×10^9^ (5 mice), or 1×10^8^ (5 mice) particles of A/NP-rAd. 4 weeks after immunization, animals were challenged i.n. with 104 TCID50 (100 LD50) A/FM/1/47-ma (H1N1) and monitored for survival (A) and weight loss (B). (0.57 MB TIF) [file pone.0013162.s004.tif]
